# Teaching complementary and alternative medicine in undergraduate medical education: a scoping review

**DOI:** 10.5116/ijme.60e2.f3ed

**Published:** 2021-07-27

**Authors:** Mary Soliman, Justin Bilszta

**Affiliations:** 1 Department of Medical Education, Melbourne Medical School, University of Melbourne, Australia

**Keywords:** Complementary and alternative medicine, undergraduate medical education, medical students, curriculum design, evidence-based medicine

## Abstract

**Objectives:**

This scoping review explores the extent to which undergraduate medical education have incorporated complementary and alternative medicine in their curricula and evaluates the teaching, delivery and assessment approaches used.

**Methods:**

ERIC, Ovid Medline and Pubmed databases were searched with keywords related to “complementary and alternative medicine” and “undergraduate medical education” for relevant articles published until August 2020. Data extraction included the presence/absence of complementary and alternative medicine integration, program duration, instructor background, and assessment methods.

**Results:**

Of 1146 citations, 26 met the inclusion criteria. Complementary and alternative medicine teaching in undergraduate medical education was widely inconsistent and not well aligned with clearly identified aims and objectives. Various complementary and alternative medicine disciplines were taught, demonstrated or observed, and several programs included teaching on evidence-based medicine. Educational outcomes mainly assessed student satisfaction and learning through self-evaluation and rarely assessed for effectiveness with regards to changing clinical practice or impacts on patient outcomes.

**Conclusions:**

Inconsistencies in complementary and alternative medicine teaching and assessment in undergraduate medical education reflect the lack of defined graduate competencies. An evidence-based medicine component of an educational program is a potential solution to overcoming breadth and content challenges. Curriculum developers would be better guided with research that determines if complementary and alternative medicine program design, content and assessment influence clinical practice and/or patient outcomes.

## Introduction

Medical educators encounter various challenges incorporating complementary and alternative medicine (CAM) teaching into undergraduate medical education (UGME). The term CAM relates to the use of non-mainstream practices, either together with conventional medicine (complementary), or in place of it (alternative).^1^ Common complementary health approaches include the broad descriptors of natural products (herbs, vitamins, minerals, probiotics), and mind and body practices (yoga, chiropractic, osteopathic manipulation, meditation, acupuncture, breathing exercises). In addition to the enormous breadth of CAM, there exists a degree of uncertainty around the validity and efficacy of many widely used therapies.^2^ Whereas conventional western medicine defines ‘best practice’ based on empirical trials utilising large patient populations, many CAM therapies boast an individualised approach where the practitioner-patient interaction is therapeutic rather than the therapy itself.^2^ This poses a considerable challenge for educators tasked with determining the necessary acquisition of CAM knowledge and skills in UGME.

Despite a need to upskill medical students in CAM-related knowledge and practice, there has not been an authoritative consensus regarding the acquisition of CAM skills and knowledge in medical students at graduation or the optimal method to provide this education. This raises the question as to what evidence and scholarship can curriculum developers draw upon to plan and implement CAM curricula for UGME students? A preliminary search of the literature, whilst failing to identify any reviews which have systematically investigated CAM teaching in UGME curricula, did provide some insight into the challenges (and innovations) medical schools face in teaching and assessing student learning related to CAM practices. Stratton and Colleagues (2007) surveyed CAM educational programs funded by the National Center for Complementary and Alternative Medicine (NCCAM). They found an array of curricula exist to provide health professions students with the necessary knowledge, attitudes, and skills to address CAM-related issues, and the approaches to evaluating curricular efforts were equally diverse; limiting the survey to only those that received NCCAM funding means it is difficult to generalise the findings.^3^ The second attempted to systematically evaluate evidence of effective CAM educational interventions for both biomedical doctors and medical students.^4^ This review only focused on descriptions of randomized controlled trials (RCTs), nonrandomized controlled trials (non-RCTs), and before and after studies. Importantly, it did not specifically examine descriptions of integrating CAM materials in the broader context of UGME curricula, nor did it explore the variety of teaching, learning and assessment approaches UGME programs utilise. This latter issue is of particular interest as it is important to determine if there is a correlation between changes in student’s CAM-related attitudes, knowledge, skills and the provision of patient care.

We, therefore, conducted this scoping review of primary studies to evaluate the different approaches UGME programs have taken to incorporate CAM teaching into their curricula and identify directions for future research. The specific research questions we sought to answer were: 1. do UGME programs teach students about CAM and, if so, which CAM disciplines do UGME programs teach students? 2. what teaching and learning approaches do UGME use to teach students about CAM? and; 3. how are UGME students assessed about their knowledge of CAM? In addition to these questions, this review also evaluated the effectiveness of CAM teaching in the included studies, using the Kirkpatrick Hierarchy for Assessing Educational Outcomes.^5^^-^^7^

## Methods

This study adopted the “Preferred Reporting Items for Systematic reviews and Meta-Analysis extension for Scoping Reviews” (PRISMA-ScR) reporting protocol.^8^

### Search Strategy

Electronic databases ERIC, Ovid Medline and PubMed were searched for full-text articles describing the delivery of CAM teaching in UGME (see Table 1). Additional papers were found through a hand search of the reference lists of articles identified through the online database search.

### Inclusion and Exclusion Criteria

There was no limit on the publication date. Only full-text articles written in English were included. Articles were limited to those that looked exclusively at UGME, with medical students only. Articles exploring medical students’ or faculty members’ attitudes regarding CAM were excluded. Articles that did not clearly describe the characteristics of CAM teaching within the UGME curricula (for example, topics taught in CAM programs, duration or frequency of teaching programs, methods used to assess student learning, etc.) were also excluded, as were general articles about CAM in medical education, and proposals for CAM curricula without implementation and opportunity for subsequent evaluation.

**Table 1:** Keyword search strategy with combined search terms

No	Keyword Search Strategy
1	(Complementary and alternative medicine or CAM or complementary medicine or alternative medicine or homeopath* or naturopath*)
2	(Medical school or medical education or education or teach* or undergraduate medical or curricul* or course*)
3	1 and 2
4	(Medical school or medical education or medical program or medical curricul* medical course* or undergraduate medical or cirricul* or course*)
5	1 and 4

### Key terms and Boolean Operators

Complementary and alternative medicine, CAM, complementary medicine or alternative medicine, homeopath*, Chinese medicine, undergraduate medical, medical school, medical education, medical course, medical cirricul*, teach, university.

### Data Extraction and Charting

Data extraction was performed using a predetermined list, and included:

·            Article details: first author and publication year

·            Participant information: student cohort, institution, or country/city of institution

·            Information gathering approach: questionnaire, survey, telephone interview

·            Educational intervention: presence or absence of CAM integration, topics taught in CAM programs

·            Duration or frequency: time spent delivering teaching within the program

·            Instructors background: qualifications or title of educators

·            Outcome assessment: methods used to assess program aims, primarily students’ qualitative and quantitative course evaluation

·            Educational outcome: assessed using Kirkpatrick’s Hierarchy of Educational Outcomes, a well-recognised tool for the evaluation of the effectiveness of medical, educational outcomes.^5^^-^^7^ The bottom level assesses learners' satisfaction with, or reaction to, the Intervention; the second level assesses modification of students’ attitudes and perceptions and/or the knowledge and skills learned; the third level assesses changes in health professionals' behaviour or an institution's practice, and; at the top of the hierarchy, changes in patient health care outcomes. In this review, Kirkpatrick’s Hierarchy was used to assess educational interventions as they relate to specific CAM programs taught within UGME curricula.

### Limitations: identified program limitations

#### Synthesis of Results

Each of the included studies was described by the author, year of publication, and the characteristics listed above. Thematic analysis was conducted to identify commonalities between the included studies. No inferences were made about CAM teaching, learning, and assessment approaches if they were not explicitly stated.  Literature searching, title and abstract screening, full-text review and data extraction and charting were undertaken by the first author (MS). Where there was any uncertainty regarding the aforementioned, these articles were reviewed independently by the 2nd author (JB) and then discussed until consensus was reached between both authors. The 2nd author also independently reviewed the data extraction and charting results once this process was completed by the first author.

## Results

**Figure 1:**
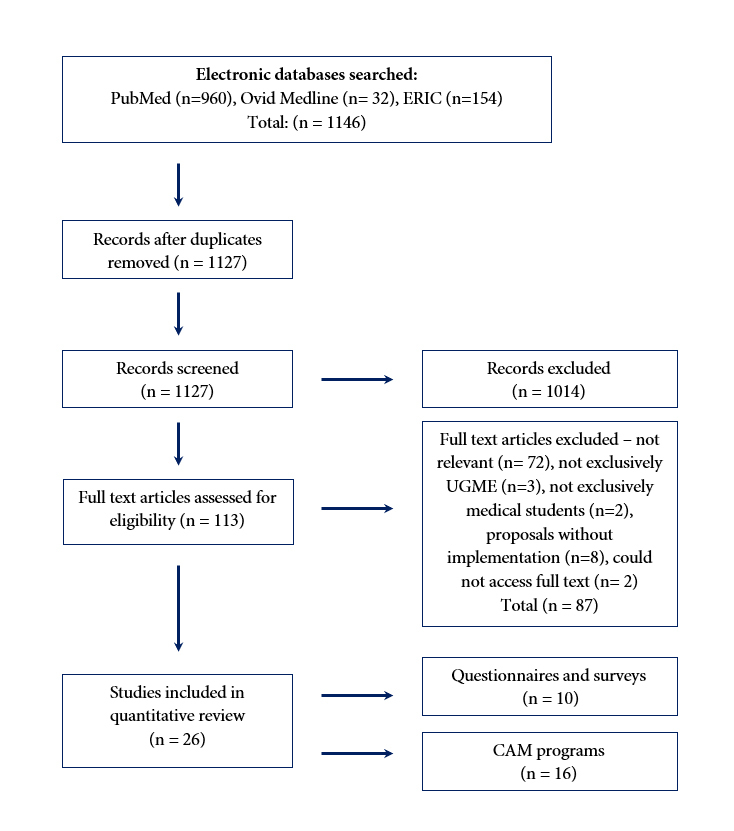
PRISMA Diagram

The primary search, which was conducted between July-September 2020, yielded 1146 citations - 960 citations from PubMed, 32 from Medline and 154 from ERIC. After the removal of duplicate citations and those not written in English, 1127 remained. Following a review of titles and abstracts, 113 full-text articles remained, of which 26 met the inclusion criteria. See Figure 1 for the complete search and study selection strategy. A summary of the included studies is found in Table 2 and Table 3. Two main categories of literature were identified: 1. those reporting whether CAM was included in a medical school’s UGME curricula, and; 2. those describing the teaching, learning and assessment approaches implemented in UGME.

### Is CAM Being Taught in UGME?

Ten studies directly addressed the inclusion of CAM in UGME curricula (see Table 2). Seven studies^9^^-^^15^ utilised written questionnaires or surveys to collect program information, whilst two^16^^, ^^17^ used a telephone interview, and one ^18^ used a combination of both. Sampson and Colleagues^13^ and Brokaw and Colleagues^10^ assessed CAM education in U.S. medical schools. Sampson and Colleagues reported 45% (56/125) of medical schools offered some form of CAM education. Brokaw and Colleagues reported of 53 U.S medical schools surveyed, 75.3% (40/53) taught an elective CAM course, and 30.1% (16/53) taught a required course, with several schools (7/53) offering both. In neighbouring regions of North America, Ruedy and Colleagues^17^ found of the 16 Canadian medical schools evaluated, 81% (13/16) reported the inclusion of CAM in their medical programs, and the remaining 19% (3/16) were planning to include CAM teaching in the future.  Rampes and Colleagues^12^ survey of British medical schools reported only 3 of 24 schools (12.5%) offered CAM teaching, and none provided practical training. Smith^14^ found CAM was included in the curricula of all 18 responding UK medical schools (58% response rate), with 33.3% (6/18) indicating it was taught formally within the UGME curriculum.

In Europe, Brinkhaus and Colleagues ^9^ surveyed 487 department directors at medical schools in Austria, Germany and Switzerland. Of these, 34% (162/487) indicated CAM had been integrated into their medical schools’ curricula. There was noted inter-country variability with lower CAM integration in Switzerland (20%) compared to Austria (28%) and Germany (27%). Varga and Colleagues^15^ surveyed 265 medical faculties in European medical schools, reporting that 40% of responding universities offer some form of CAM teaching.  In Japan, Tsuruoka and Colleagues^18^ reported CAM teaching was included in 16 of 80 (20%) schools, with 19 different teaching and learning approaches described. Kim and Colleagues (2012) reported CAM was taught at 85.4% (35/41) of participating Korean medical schools.^11^ Chitindingu and Colleagues^16^ survey of seven South African schools reported one school was teaching both Traditional Medicine (T.M.) and CAM, five were teaching either T.M. or CAM, and one was teaching neither. T.M. relates to therapeutic practices that incorporate plant, animal and mineral-based medicines, spiritual therapies, manual techniques and exercises, often practiced in developing countries.^19^ Whilst TM is regarded as distinct from CAM, TM practices may be included within CAM.^20^

Data on what elements of CAM are taught in UGME was inconsistently reported. In general, of studies that looked at whether CAM was taught in UGME, acupuncture was the most frequently taught modality^11^^,^^12^^,^^17^^,^^18^ followed by homeopathy^10^^-^^12^^,^^17^ and manipulation or chiropractic therapies.^10^^,^^12^ In studies evaluating how implemented CAM programs are taught, the most commonly taught or demonstrated CAM disciplines were acupuncture^21^^-^^25^ manipulation/chiropractic ^24^^,^^26^^, ^^27^ and massage.^21^^-^^23^ Less frequently taught therapies included biofeedback^21^^,^^26^ nutrition^21^^,^^28^ analysis of medicinal plants^24^ homeopathy^26^^,^^27^ hypnosis^26^ and osteopathy.^27^ Several of the identified programs taught about the evidence base of CAM alongside the risks and benefits or offered an opportunity for scientifically evaluating CAM efficacy.^21^^,^^24^^,^^27^^, ^^29^^-^^32^

### How is CAM Taught in UGME?

Sixteen articles^21^^-^^30^^,^^32^^-^^37^ described specific CAM teaching, learning, and assessment approaches in UGME curricula. A detailed breakdown of the different approaches used is described in Table 3. The structure of teaching modules varied greatly between programs. In a unique approach, Da Silva and Colleagues^22^ reported a split teaching program, where students attended CAM classes in their third year, followed by clinical placements in their fifth year. Meanwhile, other programs included integrated CAM teaching across three ^28^ four ^24^^, ^^34^ five^30^ and six years^25^ of their UGME programs. Other programs adopted a block approach, where teaching was delivered over a period of weeks to months.^27^^-^^29^^, ^^31^^, ^^35^^, ^^36^

Teaching time also varied greatly across institutions. Duration of time ranged from a relatively brief 5 hours^21^, to the longest of 90 hours.^25^ Several of the programs with integrated teaching across several years did not report on the total teaching time, including Frenkel and Colleagues^34^ and Perlman and Stagnaro.^24^ The teaching modalities used in the delivery of CAM education varied widely. Only one program^24^ used a solely didactic approach. Others used a didactic approach in conjunction with some form of interactive teachings such as tutorials^24^^, ^^30^ hands-on practice^23^ discussion-based learning ^31^^,^^35^^,^^36^ workshop^34^ case and team-based learning^28^ or student-led presentations.^35^ In a commonly utilised approach, several programs included a clinical placement in combination with formal lectures or tutorials.^22^^,^^25^^-^^28^^,^^31^ These placements varied in length from one day^26^ to four-afternoon sessions of unspecified duration^22^ to 30 hours.^25^ Bailey and Colleagues^21^ reported a unique approach involving a seminar followed by an Integrative Medicine fair where over 30 providers interacted with students through a series of student-selected workshops that introduced fields such as nutrition, massage, acupuncture, yoga and biofeedback. Similarly, the program described by Lehmann and Colleagues^35^ offered a unique one-day excursion to the European Library for Homeopathy (Kothen, Germany).

**Table 2:** Is CAM being taught in UGME?

Reference	Participants	Information Gathering Approach	Result / Conclusion
Brinkhaus and Colleagues 2011^9^	1,017 department directors at medical schools in Austria, Germany, and Switzerland. 487 questionnaires (response rate: 48%, country-specific response rate: A 39%; G 49%; S 42%) were returned.	Standardised questionnaire	162 respondents (34%) indicated that CAM therapies had already been integrated into the curriculum (treatment 26%, research 19% and education 18%) with no significant differences between the countries. Respondents of Switzerland indicated lower activity of CAM integration (treatment 10% and research 10%) compared to Austria (28%, p = 0.016 and 28%, p = 0.016) and Germany (27%, p = 0.01 and 20%, p = 0.174).
Brokaw and Colleagues 2002^10^	123 CAM course directors at 74 U.S. medical schools. Questionnaires were returned by 73 course directors at 53 schools.	Questionnaires mailed to course directors. The 2 page questionnaire consisted of nine questions with a check-box or fill-in-the-blank format, and one space at the end for written comments.	75.3% (40/53) taught an elective CAM course, and 30.1% (16/53) taught a required course. Topics most often being taught were acupuncture (76.7%), herbs and botanicals (69.9%), meditation and relaxation (65.8%), spirituality/faith/prayer (64.4%), chiropractic (60.3%), homeopathy (57.5%), and nutrition and diets (50.7%). Amount of instructional time varied widely, but most received about two contact hours. The "typical" CAM course an elective, was most likely to be taught in the first or fourth year of medical school, and had fewer than 20 contact hours of instruction. Most of the courses (78.1%) were taught by practitioners or prescribers of CAM therapies. Few of the courses (17.8%) emphasized a scientific approach to the evaluation of CAM effectiveness.
Chitindinguand Colleagues 2014^16^	Heads of School from seven South African medical schools	Telephone survey	One school was teaching both Traditional African Medicine (T.M.) and CAM, five were teaching either T.M. or CAM and another was not teaching any aspect of TCAM. Conclusion: Medical schools have not responded to government policies or contextual realities by incorporating TCAM into the curriculum for their students.
Kim and Colleagues 2012^11^	Academic or curriculum deans and faculty at each of 41 Korean medical schools. Replies were received from all 41 schools.	A mail survey was conducted from 2007 to 2010.	CAM was taught at 35 schools (85.4%). Most common courses were introduction to CAM or integrative medicine (88.6%), traditional Korean medicine (57.1%), homeopathy and naturopathy (31.4%), and acupuncture (28.6%).
Rampes and Colleagues 1997^12^	24 of 26 Deans of British medical schools responded	Questionnaire	Of 24 medical schools, 3 were offering teaching, and none were providing practical training. Acupuncture is included in the curricula of all three of these schools, and hypnosis, homoeopathy, manipulation and therapeutic massage in two.
Ruedy and Colleagues 1999^17^	16 Canadian undergraduate medical schools deans or faculty members.	Telephone interview lasting approximately 30 minutes was conducted with most respondents.	Most schools reported that they include CAM in their curricula (13/16), usually as part of a required course. Lectures constitute the most frequent method of information delivery, predominantly during the preclinical years. Acupuncture (in 10 schools) and homeopathic medicine (in 9 schools) were the interventions most often included. Only 2 schools reported that they provide instruction on the actual practice of one or more complementary therapies.
Sampson 2001^13^	Survey of 125 U.S. medical schools	Questionnaire to learn of approaches to CAM in curricula.	Of the 56 schools that had some form of relevant course offering, only nine had invited critical lecturers on occasion; their courses were otherwise generally supportive of CAM. Two course directors claimed to present information “neutrally,” but did not teach critical methods or invite critical lecturers. Only four courses either presented a critical orientation or offered critical arguments in a way that significantly investigated advocacy arguments.
Smith 2011^14^	Deans of U.K. Undergraduate Medical Schools. The overall response rate was 58.1% (18/31).	Survey	All respondents indicated that their curricula included CAM elements. However, the quantity of CAM within curricula varied widely between medical schools, as did the methods by which CAM education was delivered. General Medical Council requirements were the strongest factor influencing the inclusion of CAM, although medical student preferences were also important. Respondents were generally satisfied with the extent of CAM provision within their curricula, while a wide range of views on the appropriateness of CAM in the medical curriculum were held by faculty members.
Tsuruoka and Colleagues 2001^18^	80 Japanese medical schools for Western medicine. Response rate to the telephone survey and self-completed questionnaire was 100 and 95%, respectively.	1. A telephone survey to curricular office workers in September 1998 2. A self-completed questionnaire to representatives of sponsoring departments	Of 80 medical schools, CM was officially taught in 16 schools (20%). Of these 16 schools, there were 19 CM courses and the anesthesia department sponsored the most courses (six courses). All courses had oriental medicine titles such as acupuncture and Kampo except for one course.
Varga and Colleagues 2006^15^	265 medical faculties in E.U. countries were contacted via e-mail or regular post	Questionnaire of 7 questions concerning CAM education in their establishments.	Only 40% of the responding universities were offering some form of CAM training. Could not show any correlation between the public demand for CAM methods and the availability of CAM training in medical universities.

Generally, the instructors delivering CAM programs were either faculty members of the medical school or clinicians^29^^,^^30^^,^^34^^-^^36^ external CAM practitioners only^26^^,^^31^ or both.^21^^,^^24^^, ^^25^^,^^27^^,^^32^ Several programs also included visiting experts and scholars from other universities.^24^^,^^28^  In some instances, faculty members were noted to have undergone advanced CAM training.^24^^,^^32^ Several programs involved qualified community or student CAM providers and practitioners, including those with an element of clinical placement.^21^^,^^23^^,^^26^^,^^27^^,^^31^ Post course student evaluations were infrequently used to assess student experiences and overall course satisfaction.^26^^,^^31^ Objective assessment of knowledge and learning was not performed. Where knowledge assessment was completed, it often entailed subjective self-reporting using a Likert scale or other quantitative measure.^22^^,^^23^^,^^27^^-^^29^^,^^36^ Few programs used appropriately coded and thematically interpreted qualitative measures.^34^^,^^35^ Several programs did not have formal evaluative or assessment processes in place^24^^,^^25^^,^^30^^,^^37^ or removed the evaluative process following a period of time.^21^

### Assessment of Education and Learning Outcomes

This review assessed the educational and learning outcomes of the aforementioned studies using Kirkpatrick’s Hierarchy (Table 2).^5^^-^^7^ There was a clear trend toward Level 1 outcomes, where student reaction and satisfaction were measured in all programs, bar one^34^ using a post-course evaluation. Likewise, a majority of programs achieved Level 2 outcomes by considering the achievement of learning outcomes by students who completed self-evaluations.^21^^,^^22^^,^^27^^-^^29^^,^^32^^,^^34^^-^^36^ Only Frenkel and Colleagues^34^ achieved Level 3 outcomes, where students were asked if and how learned knowledge from the CAM program would change their behaviour. It should be noted behavioural changes were self-reported and not directly observed or analysed. Level 4 outcomes were not achieved by any of the UGME programs included in this review.

## Discussion

This scoping review of CAM education in UGME demonstrates the emerging interest in this area. In view of the rising use of CAM and the potential for CAM-conventional therapy interactions, there is an increasing need for physicians to become familiar with common CAM therapies.^38^ As reported, past and current inclusion of CAM teaching, even within schools located in the same country, is inconsistent. In curricula that include CAM, teaching and learning vary widely, both in terms of content and delivery. It appears agreed statements on the expected skills and competencies of medical students at graduation related to CAM therapies are yet to emerge. In the absence of this consensus, it is difficult to identify clear aims and objectives of any CAM teaching program within a medical course. The following discussion amalgamates the key findings and addresses their implications, with the overall aim of aiding in the development of consistent and equitable medical education.

### Program Format and Design

The duration of teaching is one of the greatest areas of inconsistency amongst the included programs. Whilst a number had integrated CAM teaching across one or several years of their curriculum, the design of others was relatively brief, with few - or unspecified - dedicated teaching hours.^21^^,^^32^^,^^37^ Meanwhile, several ‘integrated’ programs regarded the practices of mindfulness and self-care for students as an element of their holistic CAM curriculum.^23^^,^^30^ Other medical schools considered mentioning CAM in areas of relevance - such as drug interactions and clinical oncology - constituted sufficient CAM teaching.^14^ This could lead to a potentially inflated and inaccurate measure of the duration of CAM teaching, particularly within these integrated approaches.

In analysing the approach to teaching, it is clear experiential learning is favoured compared to a solely didactic approach. Nearly all programs favoured delivery methods that would enhance student engagement. These included tutorials, workshops and case discussions, alongside clinical placements with CAM practitioners. Whilst well received by students, most immersive methods only achieved Kirkpatrick’s Level 1 and/or 2 outcomes, without an assessment of translatability to clinical practice and impacts on patient outcomes, which would require Level 3 and 4 outcomes, respectively. Various programs included clinical placements with CAM practitioners, the longest being 30 hours.^25^ However, in the absence of a student evaluation or objective assessment tool, the benefits of clinical placement cannot be commented on.

Various programs focussed on specific CAM disciplines, the most common of which was acupuncture.^21^^-^^25^ This reflects the identified difficulties in establishing a discrete knowledge base, as CAM disciplines require constant updating as new evidence and novel therapies emerge and gain traction within the general population.^3^ In what proved to be a popular approach^21^^,^^24^^,^^27^^,^^29^^-^^32^ analysis of the evidence base surrounding CAM is one of the more common methods of teaching. In equipping students with the skill set needed to critically appraise evidence, the plethora of CAM therapies need not be delved into individually. Rather, students can draw their own informed conclusions without the influence of potentially biased and unsubstantiated claims. Despite this, not all programs included teaching on evidence-based medicine (EBM), rather focusing on specific CAM disciplines. In designing a sustainable CAM program, curriculum developers should consider the need for constant review and critical appraisal as new evidence emerges. The EBM teaching model presents a potential solution to a rather overwhelming and resource intensive area of education, where learned knowledge and skills can be adapted for differing CAM modalities, and beyond.

**Table 3:** How is CAM taught in UGME?

Reference	Participants	Education Approach/ Intervention	Duration and frequency	Instructors background	Outcome measure	Outcomes Assessment	Limitations
Bailey and Colleagues 2015^21^	Fourth year medical students at Duke University, USA	Initially a 90-minute interactive seminar providing introductory core learning. This developed into a seminar and I.M. fair. Included I.M. fields were nutrition, massage, acupuncture, yoga and biofeedback. The program changed over time from assigned to student selected teaching modalities.	1 hour seminar + 4 hour fair during which each student attended 4 x 40 minute workshops. This structure was adapted and repeated from 2005 -2013	Duke University faculty, staff and community CAM providers	1, 2a, 2b, 3	Qualitative and descriptive data from course evaluations completed by students, and quantitative data from the AAMC graduate questionnaire. Pre and post knowledge testing was completed for an unspecified period of time, then removed.	Removal of pre-post knowledge testing
da Silva and Colleagues 2013^22^	Third and fifth year medical students at Rio Preto Medical School. Brazil	Third year students receive acupuncture classes as part of their formal curriculum. Fifth year students attend acupuncture outpatient clinics	Third year: 6 hours of classes Fifth year: 4 x afternoon sessions in clinic	Not specified	1, 2a, 2b, 3	5 question, 1-5 scale evaluation completed by 2011 and 2012 cohort. Subjective assessment of knowledge and likelihood of identifying when CAM is suitable for patients.	Lack of objective measure of knowledge or skills
Forjuoh and Colleagues 2003^29^	Third year medical students of a state, public medical school in Texas, USA	Interactive educational sessions on family medicine, with CAM teaching integrated within. In addition, EBM was taught as a tool to teach CAM, enabling students to critically appraise therapies for their safety and efficacy in clinical practice.	5 hour clerkship session x 6 weeks	Faculty members including the director of research and a family physician	1, 2a, 2b	Likert scale and Wilcoxon signed rank test used in a pre and post-curriculum questionnaire evaluating changes in students perceived knowledge, attitudes, and skills.	Lack of objective measure of knowledge or skills, and small sample size, further reduced by 19% that did not complete the evaluation.
Frenkel and Colleagues 2007^34^	4 graduates from the School of Medicine, University of Texas, USA who had completed 4 years of the CAM project	The curriculum had included multiple multidisciplinary lectures, workshops, electives and structured rotations and additional educational activities.	Integrated teaching across 4 years	Two family medicine physicians	1, 2a, 2b, 3	Qualitative in-depth, face to face, semi-structured interviews – coded and thematically interpreted	Very small sample size and interviewer bias
Hassed 2004^30^	Undergraduate medical students at Monash University, Australia learning integrated CAM over the 5 year duration of the medical degree.	Core curriculum teaching including mindfulness-based stress management programs, lectures and forums on complementary medicine, and integration into weekly case-based teaching. This covers principles, research, evidence base, ethics and clinical applications. Additional opportunities to undertake optional CAM electives offered.	3 day transition camp with self-care theme, 2 introductory lectures, 6 x 2 hour tutorials, 2 mind body medicine lectures, 8 hour CAM teaching in second year Optional elective: 12 weeks x 2 hours	Not specified	N/A	Nil	Overview of ‘holistic’ nature of the medical program lacked student perspectives and outcomes assessment
Hoffmann and Colleagues 2019^23^	40 first-year medical students at University of Iowa Carver College of Medicine, USA	The experimental group viewed educational videos and participated in hands-on massage practice The control group only viewed the educational videos.	Hands on massage x 11 hours and/or Educational videos x 4 hours, over 6 week period	Two co-course directors and five current massage therapy students	1, 2a, 2b	WHO Quality of Life Bref Survey, 6-8 students from both groups participated in post-course focus groups, knowledge assessment using non-validated tools. Participants completed pre- and post-course assessments of knowledge, attitudes, and personal wellness	Small sample size Some non-validated assessment tools
Jeffries 2001^31^	Unspecified sample size of medical students from Creighton University School of Medicine, USA	Senior elective with lectures and group discussions. Included conducting research on CAM, a scientific evaluation of efficacy, and a clinical rotation.	4 week duration	CAM practitioners supervising clinical rotation	1	Post-course survey assessing student satisfaction	Unknown sample size Lack of pre-evaluation Lack of objective outcome measure
Karpa 2012^28^	23 fourth year medical students from Pennsylvania State University College of Medicine, USA	A herbal/natural product course using classroom presentations and active learning mechanisms that include experiential rotations, case-based learning, and team-based learning.	40 classroom hours, and clinical rotations. The course was carried out annually over 3 academic years	Pharmacology faculty member, multidisciplinary faculty members and guest lecturers with varied backgrounds	1 & 2a	Final course grades determined on the basis of in-class presentations, attendance, participation and professionalism in class and clinical rotations. Likert-type questions and narrative responses used to assess student opinion of knowledge and skills imparted by the elective and overall course content	Limited enrollment capacity and scheduling difficulties reducing the sample size Inability to directly measure the impact that the course has had on student-patient interactions in clinical encounters
Laken and Cosovic 1995^26^	Seven medical students electively enrolled from Wayne State University Medical School, USA	Senior elective delivered using didactic lecture, films, first-hand experience and observation of alternative practitioners. Students explored hypnosis, chiropractic, therapeutic touch, medication, biofeedback, acupuncture, homeopathy, naturopathy, and massage therapy.	7 days of formal teaching 1 day observation clinical placement	Alternative medicine practitioners in the Detroit area	1	Student evaluation of course structure and content	Lack of objective assessment of knowledge
Lehmann and Colleagues 2014^35^	30 medical students at the Institute for General Practice and Family Medicine at the Otto Von Guericke University, Germany	Elective course involving introductory lectures followed by discussion, performance of practical exercises, and student presentations on a self-chosen topic. Also included a one day excursion to the European library for homeopathy (Kothen).	Three weekend course OR a block course – total of 56 hours in either form	Conventional medicine practitioner	1, 2a, 3	Semi-structured discussions for a qualitative analysis. Topics included experience of the seminar, and anticipated use of homeopathy in future practice.	Voluntary participation in an optional subject may have lead to less critical, more positive results on the survey, Small sample size of students, bias
Ma and Colleagues 2014^36^	251 students at a Chinese Military Medical University, China	EBM course formally included in the curriculum, combining lectures with small group discussion and student-teacher exchange sessions. It included 5 lectures and 2 seminars.	20 hour course	Faculty staff	1 & 2a	Pre and post training surveys with comparisons of percentage change of scores pre and post training using 6 point Likert scale	
Mahapatra and Colleagues 2017^32^	17 students (33%) in the class of 2015 and 22 students (42%) in the class of 2016 from Mayo Clinic School of Medicine, USA	A mandatory short I.M. curriculum across all years of medical school. Content focused on basic science and experimental and evidence based knowledge.	Not specified	I.M. professionals and physician faculty members with expertise in integrative therapies.	1 & 2a	Paired data analysis of students who completed two surveys in their first and third year. Chi-square test, Wilcoxon rank sum test, McNamara agreement test, signed rank test used.	Lack of objective measure of students knowledge
Maharaj 2010^37^	Selective modules Retrieved to 24/ 160 students from University of the West Indies, Jamaica	Assessing spiritual health and an introduction to alternative medicine practices	Not specified	Not specified	1	No formal evaluation, positive responses expressed in writing by students	No formal evaluation of the program
Owen anLewith 2001^27^	Unspecified number of undergraduate medical students at Southampton University, U.K.	Optional modules addressing the issues raised by CAM, and examining its evidence base. Covered CAM therapies included homeopathy, chiropractic, osteopathy and acupuncture. Additional local clinic attachments in both NHS and private practice	8 session module, repeated bi-annually, over a 3 year period	Three doctors	1 & 2a	Subjective student questionnaire with Likert-based format + Written comments encouraged	Lack of pre and post intervention knowledge measure
Perlman and Stagnaro-Green 2010^24^	New Jersey Medical School at the University of Medicine and Dentistry, USA	Evolution of a complementary, alternative, and integrative medicine course with clearly stated core competencies and goals. The program included lectures and demonstrations of acupuncture and manipulation. Included teaching about appraising evidence and the ethical issues raised, and proposed a clerkship in 3^rd^ and 4^th^ year.	4 year integrated teaching	Faculty members with advanced training or knowledge of CAM, including a faculty member from the local massage school	N/A	Not specified	No formal evaluation of the program
Tahzib and Daniel 1986^25^	Unspecified number of undergraduate medical students at the University of Sokoto, Niger	Lectures, tutorials, seminars demonstrations of techniques such as acupuncture, practical exercises in analyzing medicinal plants, and supervised field visits to high grade traditional medical practitioners.	90 hours over 6 years: 60 hours of content, 30 hours of clinical placement	Academic medical staff, visiting experts, scholars from other universities, traditional medical practitioners	N/A	Not specified	No student evaluation Lack of objective knowledge assessment

Likewise, the qualifications of CAM educators for such programs must be consistent and their repeated involvement sustainable. Whilst many programs utilised community-based CAM practitioners^25^^,^^26^^,^^31^^,^^35^ difficulties in repeatedly sourcing appropriately qualified instructors were identified.^21^ Where faculty staff were involved in teaching, several programs were sustainable for longer.^21^^,^^22^^,^^30^ Whilst both CAM practitioners and faculty staff could both introduce an element of bias, this could be overcome with clear learning outcomes and an evidence-based teaching approach. In view of sustainability, it may be more appropriate to deliver teaching through faculty staff, some of who receive further training in EBM. Curriculum developers should consider and account for the additional cost of incorporating CAM programs within UGME.

### Appropriate assessment and educational outcomes

Few of the programs performed formalised pre and/or post-course student assessment or evaluation as a measure of the change in knowledge. This reaffirmed findings from Stratton and Colleagues^3^ which reported the same observation in the programs receiving CAM education grants from the NCCAM. Instead, general qualitative statements were regarded as a measure of overall student satisfaction and increased knowledge.^21^^,^^27^^,^^34^ Student reactions and knowledge (Kirkpatrick’s Level 1, 2a, 2b) were the most commonly achieved outcomes, with student knowledge measured subjectively through student questionnaires.^22^^,^^27^^-^^29^^,^^31^^,^^32^ Whilst Frenkel and Colleagues^34^ was successful in determining the willingness of learners to apply new knowledge and skills (Kirkpatrick’s’ Level 3 outcome), it was the only study to do so. Course designers within medical schools must create educational programs that aim to directly impact patient care, rather than increasing knowledge without a foreseeable change to clinical practice. This is an undoubtedly challenging task, particularly since many widely taught CAM therapies have not yet been proven efficacious by scientific standards. For medical educators to educate appropriately, an evidence base for positive patient outcomes must first be established.

### Limitations

Articles may have been omitted due to the adopted search strategy, inclusion criteria, and limit of English language articles only. Grey literature was not performed. Several programs were incompletely reported, potentially influencing the outcomes reported in this review.  Many medical school faculty and Deans who were approached did not participate, leading to potential selection bias. Studies incorporating face-to-face interviewing may also be subject to observation bias. A small sample size, apparent in several studies, could also reduce the power and, therefore, reliability of results. Post-evaluations using subjective qualitative Likert-based assessment do not provide an objective measure of program success, particularly where students’ results cannot be compared to pre-program standards. As only the first three levels of Kirkpatrick’s Hierarchy were reached, the patient implications of CAM in UGME could not be determined. No studies reported on the change in patient outcomes or healthcare delivery (Kirkpatrick’s Level 4). Longitudinal prospective studies would provide curriculum developers with insight to the real-world effects of CAM education, where patient outcomes can be correlated to teaching interventions. Several studies did not report outcomes of interest, such as teaching duration, disciplines and teaching staff.^22^^, ^^27^^, ^^28^^, ^^31^^, ^^32^^, ^^37^

## Conclusions

The aim of this review was to evaluate the various approaches for teaching CAM in UGME. Despite various limitations, it is apparent CAM teaching is inconsistently incorporated into medical schools at a multi-national level. The diversity in approaches reflects the lack of defined graduate competencies as they relate to this specific area. With a breadth of CAM disciplines and an array of teaching and learning approaches, there is no single recommended education program that has been demonstrated to produce positive patient outcomes. Although the concept of an EBM course appeals as a potential solution to overcoming the enormous breadth and content developments in CAM, ultimately there is a deficiency of evidence to demonstrate the real-life healthcare impact. Curriculum developers would be better guided with further research, aligning health outcomes with teaching, assessment and evaluation of proposed CAM programs.

### Conflict of Interest

The authors declare that they have no conflict of interest.
